# The effects on oxidative aging, physical and flow properties of Agbabu natural bitumen modified with silver nanoparticles

**DOI:** 10.1016/j.heliyon.2020.e04164

**Published:** 2020-06-27

**Authors:** Ojeyemi M. Olabemiwo, Agbaje Lateef, Foluso O. Agunbiade, S.B. Akanji, Hassan O. Bakare

**Affiliations:** a Department of Pure and Applied Chemistry, Ladoke Akintola Universityof Technology, Ogbomoso, P.M.B. 4000, Nigeria; b Laboratory of Industrial Microbiology and Nanobiotechnology, Department of Pure and Applied Biology, Ladoke Akintola Universityof Technology, Ogbomoso, P.M.B. 4000, Nigeria; c Department of Chemistry, Faculty of Science, University of Lagos, Lagos, Nigeria; d Department of Science Infrastructure, National Agency for Science and Engineering Infrastructure Idu Industrial Layout, Abuja, Nigeria

**Keywords:** Nanotechnology, Physical chemistry, Natural bitumen, Carbonyl and sulphoxide peaks, Silver nanoparticles, Physical and flow parameters, Antioxidants, Ageing

## Abstract

The quest for improvement in service life and performance of road pavement via reduction of oxidative aging failure of bitumen, led us to the investigation of novel application of Silver nanoparticles (AgNPs) as potential anti-oxidative material for Agbabu natural bitumen (ANB). The raw ANB was purified to form the base and the base modified in a stainless reactor using AgNPs via melt blend technique at temperature of 120 °C under stirring at 1200rpm. The proportions of AgNPs used for the modification were 1.5, 3.0 and 4.5 wt% and long-term aging was thermally simulated on the base and modified base samples at 60 °C. The aged samples were then subjected to Fourier Transform Infrared (FTIR) Spectroscopic Analysis to study the changes in the size of the peaks of the oxidation-related compounds. Physical and flow parameters (PFPs) of the base and modified base samples were characterized using softening point temperature, kinematic viscosity, penetration index, flash and fire points, penetration, kinematic viscosity and Oscillatory disc Rheometer (ODR) test. FTIR analysis showed that the AgNPs incorporation into ANB at 1797 cm^−1^, 1217 cm^−1^, 1300 cm^−1^ and 1097 cm^−1^ in the spectrum of the base sample. The sulphoxide peaks at 1031 cm^−1^ was completely obliterated. There was progressive reduction in the area of the carbonyl peak at 1693 cm^−1^ implying progressive lowering of the carbonyl index value with increasing in the amount of AgNPs used in the modification. These changes are attributable to the anti-oxidative potential of the AgNPs. The mechanism of the anti-oxidative effect of AgNPs is proposed to be due to scavenging of the free radical produced in the oxidation process. The values of softening point temperature, kinematic viscosity, penetration index, and flash and fire points increased while that of penetration and specific gravity reduced as the quantity of AgNPs in the base increased. The ODR test showed that, the modified samples compared to base sample at lower and higher road pavement temperatures are less prone to fatigue cracking and rutting, respectively. Thus, this study provides preliminary information about the novelty of AgNPs as potential antioxidant for improving the durability/performance of bitumen in pavements.

## 1 Introduction

Rapid deterioration of bitumen pavements which reduces bitumen service life as binder is often caused by oxidative aging [1]. This is the major factor responsible for undue hardening of bitumen. Oxidative aging of bitumen do occur by the atmospheric oxidation of molecules leading to the conversion of non-polar molecule into highly polar and strongly interacting functional groups containing oxygen [1]. It can either be thermally induced by heating or light induced (photo-oxidation) by exposing modified or unmodified bitumen sample to sunlight or ultra violet (UV) radiation. During storage, mixing, laying and transportation processes of bituminous mixture, bitumen was exposed to undesirable rapid aging, but slow aging process occurs during the service life of bitumen in pavement [1, 2].

The basis of oxidative aging of bitumen is associable with the different types of compound in the substance which include aromatic and hetero atomic (N, S, and O containing organic) compounds, most of which are with varying molecular size, aromaticity, and polarity [3]. The chemical functionalities in these compounds are responsible for intermolecular interactions that contribute to the behaviour of the asphalt towards oxidative aging/hardening. Some of the deteriorations suffered by asphalt pavements are cracking and permanent deformation (rutting), and the extent is a function of the nature of bitumen binder used to prepare the bitumen-aggregate mixture [4]. Asphalt cracking, to certain extent is associated with oxidative aging via thermo or light aging, due to exposure of bitumen to oxygen-rich environment, while, rutting is associated with the use of high penetration grade bitumen for pavements in hot climates [5].

Previous studies by Petersen and Harnsberger [6, 7], investigated sulphoxide and ketone formation during aging of asphalt and reported that during the oxidation spurt, some of the hydroperoxides formed from the oxidation of aromatic compounds in bitumen e.g. dihydroanthracene are likely to react with aliphatic sulphides to produce sulphoxides or decompose to produce free radicals. Initiation of the oxidation of benzylic carbon requires abstraction of active hydrogen to form a benzyl radical which then reacts with oxygen at a very low concentration to form a peroxy radical. The peroxy radicals undergo non radical self (condensation) reaction to form ketones. The most probable route to sulphoxide formation following the oxidation spurt is the reaction of alkylarylhydroperoxides with asphalt sulphides. This reaction is promoted by the presence of acidic molecules. In addition, the alkylarylhydroperoxides decomposition induced by catalytic amounts of metal ions or a basic reaction medium might be an alternate route to ketone formation. The amounts of ketones formed on non-benzylic carbon were reported to be small. However, it is conceivable that higher oxidation temperatures could favour the formation of a relatively greater amount of non-benzylic ketones [6, 7].

To understand the effects of temperature on oxidative aging, two laboratory test methods have been deployed on bitumen samples: Rolling Thin Film Oven test (RTFOT) and Pressure Aging Vessel (PAV) [8]. RTFOT was used to simulate short term aging at 163 °C to measure effects of heat and air on moving film of bitumen, while PAV is used to simulate long term aging of bitumen for the next 10 years during in service in road pavement [8, 9]. Aging processes have been reported to increase the size distribution of the large binder molecules that results in the increase of the viscosity and stiffness of the binder [10]. Poulikakos *et al.* [11], has however reported that artificial aging techniques at elevated temperature above 60 °C make the microstructure of the bitumen to disappear and may limit the obtainment of useful conclusions relevant to natural aging experiments at such temperature range. Poulikakos *et al.* [11], reported that pressure aging vessel (PAV) represented artificial aging state which is completely different from that at a temperature below 80 °C (e.g. roads). This implies that a better thermal aging temperature is that in which the microstructure of the bitumen is still present (60 °C). There are four bitumen aging mechanisms that have been documented in literature: oxidation, loss of volatiles, physical hardening and exudative hardening [12, 13].

Over the years, polymers have been used as modifiers in bitumen to reduce the effects of oxidative aging and loss of volatiles from bitumen. These modifications of bitumen involve blending of certain materials which offer complimentary and/or supplementary qualities to the base bitumen by mechanical or chemical processing, to obtain an improved binder material for any intended purpose [14]. Some of the various polymers investigated in the literature were not initially designed for bitumen modification. These include: plastomer such as polyethylene (PE), ethylene-butyl acrylate (EBA), ethylene-vinyl acetate (EVA), and thermoplastic elastomer such as styrene-butadiene-styrene (SBS), styrene-isoprene-styrene (SIS), styrene-ethylene/butylene-styrene (SEBS) [15, 16, 17].

Recently, efforts to improve anti-oxidative ability of bituminous materials for use in pavement engineering have extended beyond addition of polymers. Some nanomaterials, with particle size less than 100 nm, are now being incorporated into bitumen, and they have been found in some cases to enhance the physical and flow properties of bitumen [18, 19]. The usefulness of these nanoparticles are due to their reported higher reactivity owing to their small sizes, their stability, and other unique properties that make them also useful in industrial applications [20, 21, 22].

Nano-silica, carbon nano-tubes, nano-clay, nano-zinc oxides and nano-titanium dioxides are some nanoparticles that have been studied and reported for modification of bitumen [20], but there is no information on the use of AgNPs for bitumen modification. Moreover, previous research works have documented the application of nanomaterials as modifiers on bitumen obtained from refined petroleum source. There are however limited literature information on modification of bitumen from natural source especially from Nigerian natural bitumen. Therefore, this study reports the novel modification of the base natural bitumen sample with AgNPs in order to evaluate AgNPs anti-oxidative effects and its effects on the physical and flow properties of the bitumen sample. Lateef *et al.* [23], reported the antimicrobial and anti-oxidative activities on emulsion paint by green synthesised poly-disperse spherical-shaped AgNPs materials with size ranging from 20-80 nm produced with the capping agent obtained from pod of *Cola nitida*. Others studies have also investigated the antimicrobial/inhibitory as well as size dependent activities of green synthesized AgNPs [22, 24, 25], which implies that AgNPs, asides its anti-oxidative activities can be applied in bitumen to reduce the biodegradability of hydrocarbon compounds in the bitumen. This submission is based on earlier report by Olabemiwo *et al.*, [26]. They study the biodegradation of hydrocarbon compounds in the ANB and reported a reduction in total aliphatic and poly aromatic contents as a result of its (ANB) vulnerability to biodegradation by some bacteria strains.

It has also been documented in literature that FTIR spectrometry can be successfully used for the identification and quantification of the effects of modifiers on the aging of bitumen as well as for the evaluation of their effects on chemical and physical properties of asphalt [2, 17, 27]. The use of FTIR analysis to investigate the anti-oxidative ability of AgNPs on base in a simulated long term aging under artificial condition with an oven at 60 °C [17], the effects of AgNPs anti-aging on the physical and flow properties and the study of the type of their interactions were therefore the objectives of this study. This application may be compared with the other existing nanoparticles currently being used. The use of AgNPs for modification of bitumen from natural or petroleum source has not been reported. Therefore, this study is meant to contribute to knowledge on the novelty of anti-oxidative ability of AgNPs for the modification of bitumen for pavement applications.

## 2 Experimental

### 2.1 Materials: bitumen

The bitumen used in this study was purified ANB sample of 82 dmm penetration grade. It is a black-viscous, adhesive and elastic material. The water content of the raw ANB was found to be 11.06% as reported in our previous study [28].

### 2.2 Modifier: silver nanoparticles (AgNPs)

The green, biosynthesised AgNPs used as modifier in this study was obtained from the Laboratory of Industrial Microbiology and Nanobiotechnology, Department of Pure and Applied Biology, Ladoke Akintola University of Technology, Nigeria. The AgNPs has specific gravity of 0.00017, specific surface area of ±20 m^2^/g and size ranging from 20-80 nm with nearly spherical morphology. AgNPs, an inorganic material was considered as a suitable modifier due to its beneficial properties which include: large surface area, excellent dispersion ability, high absorption capacity, excellent stability and high chemical purity.

### 2.3 Purification of ANB sample

The ANB sample collected from Agbabu was dehydrated and purified as described in our previous studies [17, 28]. Method described by Vernon and Katy [29], was modified and employed for extracting water from raw ANB, while the dehydrated sample was purified using the modified method of Rubinstein and Strausz, [30]. The raw ANB sample after purification is referred to as base sample.

### 2.4 Preparation of silver nanoparticles modified base sample

The AgNPs modified base sample was prepared using the melt blend technique in a stainless reactor. The process involved the melting of 500 g of base sample in the reactor at temperature of 120 °C under stirring at 1200 rpm. The AgNPs composition in weight percentage (wt.%) of the base sample was slowly added to the agitated base sample in the reactor, and the agitation was allowed for 1 h to produce homogenous mixture. The proportions of modifier used for the modification are given in Table 1.

**Table 1:** Proportions of modifier used for the modification of base samples.

Modified Sample	AgNPs (wt.%)
1	1.5
2	3
3	4.5

### 2.5 Fourier Transform Infrared (FTIR) analysis of the base and AgNPs modified base sample

Fourier Transform Infrared (FTIR) Spectrophotometer FTIR-8400s, SHIMADZU, with spectral range: 4,000-400 cm^−1^ was used. Samples were prepared using the method of Olabemiwo *et al.*, [17]. The IR spectra of base and the AgNPs modified base samples were generated by separately scanning each sample. Each sample (1.5 mg) was ground with 150 mg of analytical grade potassium bromide (KBr). The mixture was pressed in a hydraulic press to form a pellet. The pellet was mounted on the spectrophotometer and the infrared spectra of base and modified samples were generated by scanning.

The carbonyl index was calculated from the band areas in the spectra to determine the aging behaviour of the base sample compared to the modified samples using the equation from Mouillet *et al.* [13], in Eqs. (1) and (2):

(1) $$Oxygenated\;functions\left(carbonyl\;index\right)=\frac{A_{1700}}{\sum A};$$

(2) $$The\;sum\;of\;the\;area\;represents\;\left(\sum A\right)=A_{1700}+A_{1600}+A_{1460}+A_{1376}+A_{1030}+A_{864}+A_{814}+A_{743}+A_{724}$$

The carbonyl index values were calculated from the ratio of the area of the spectra at 1700 cm^−1^ of base and modified base samples and the areas of the peak from 700-1700 cm^−1^ as against the use of intensities [13]. This accounts for any variation in baseline as the ratio cancels any significantly error that may affect the carbonyl index values.

### 2.6 Investigation of thermal aging on base and modified base samples

This part of the investigation was carried out using the method of Olabemiwo *et al.*, [17]. Base sample (5 g) was weighed into a Pyrex glass Petri dish and spread to a thickness of 0.1 cm. The dish was then set in a hot air oven thermostatically controlled at ±1 °C. The experiment was performed at 60 °C for three weeks. Part of the sample (0.4 g) was then periodically withdrawn from the oven at interval of one week and conditioned at room temperature for IR analysis to study the sample's degradation (oxidative stress) due to heat. The choice of the oven temperature at 60 °C for three weeks as against the general PAV long term aging based on BS EN was arrived at base on Poulikakos *et al.* [11], work on the effects of high temperature and pressure. We therefore simulated a near natural, long term aging condition at 60 °C similar to the 35 days, 70 °C oven tested by Elwardany *et al.*, [31].

### 2.7 Rheological properties of base and modified base samples

Oscillating Disc Rheometer (ODR) 2000, manufactured by Alpha Technologies, was used to obtain the complex shear modulus of the thermally aged base and modified base samples using the modified method of Olabemiwo *et al.*, [17]. Twenty grams (20g) of test binder (base sample) was place in the sample chamber at a near constant temperature by heating and cooling a surrounding environmental chamber and a rotor in the middle of the chamber oscillated. The rotor is connected to a torque sensor and was oscillated through a controlled angular velocity, w (10 rad/s) to measure the torque response of the binder at deformation. This action exerted a shear strain on the test binder and the torque (force) required to oscillate the rotor was dependent upon the stiffness (shear modulus) of the binder.

The tests were carried out at low and high temperature testing with 5 °C increment. The low temperature testing was carried out at 5–25 °C to characterize fatigue cracking while the high temperature testing was carried out at 25–70 °C to characterize rutting performance. For testing temperatures at 25–70 °C, a sample chamber with a diameter of 1mm and a rotor with a diameter of 25 mm were used. While, for testing temperatures at 5–25 °C, a sample chamber with a diameter of 2 mm and a rotor with a diameter of 8 mm were used. The complex shear modulus (G∗) was calculated based on the shear strain exerted on the binder and the shear stress calculated from the maximum torque required to oscillate the rotor which was according to the dimensions of the sample chamber and rotor.

The shear stress (τ) force per unit area was calculated as shown in Equation below:

$$\text{τ}=\text{M}/2\text{π}{\text{R}}_{\text{b}}^{2}\text{L}$$

where.

R_b_^2^ = Radius of the sample chamber (cm)

L = Effective length of the rotor (cm)

M = Maximum torque input by the instrument (dynes)

While the oscillatory shear strain, γ, can be expressed as:

$$\text{γ}={\text{γ}}_{\text{0}}\;\sin\text{wt}$$

where.

${\text{γ}}_{\text{0}}$ = peak shear strain

w = angular velocity in radian/second.

The G∗ is calculated from τ/γ.

### 2.8 Physical and flow properties

The following physical and flow properties of the AgNPs modified base samples were determined using appropriate standard procedures: softening point temperature [32], Penetration value [33], Kinematic viscosity [34], Flash and fire point tests [35].

## 3 Results and discussion

### 3.1 Silver nanoparticles modified base sample

In this study, the AgNPs modified base sample was prepared at temperature up to 120 °C in order to ensure that the base sample was fully melted for proper blending of the modifier (AgNPs). It was noted that at a temperature below 100 °C, the base sample was not fully melted, thus, a temperature of 120 °C was used. Besides, the base sample was stirred at 1200 rpm having noted that the higher the rotating speed of the stirrer, the better the blending of the AgNPs with the base sample. However, a rotating speed higher than this might result in spilling or splashing of the melted sample.

### 3.2 FTIR analysis of base sample

**Figure 1:**
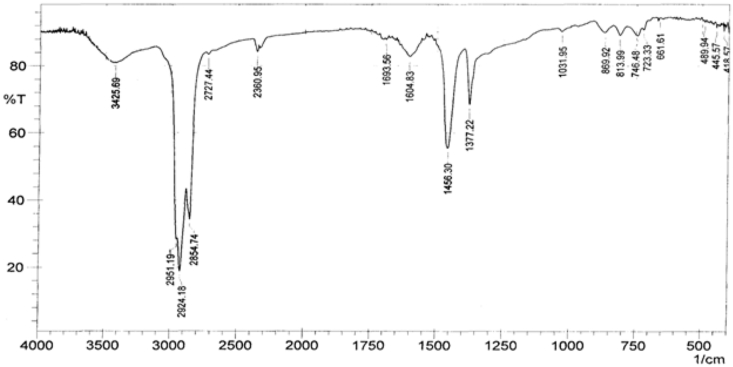
FTIR of base sample.

The FTIR spectra of base samples were recorded in the spectral range of 4000–400 cm^−1^ as shown in Figure 1. The FTIR analysis was carried out to detect the functional groups present in the base and modified base sample and to ascertain whether there were formations of new bonds via the modification process. Base sample had absorption peaks at 2924 cm^−1^, 2854 cm^−1^, 1693 cm^−1^_,_ 1604 cm^−1^, 1456 cm^−1^ and 1377 cm^−1^ and peaks in the region 1031 cm^−1^_,_ 869 cm^−1^_,_ 813 cm^−1^_,_ 746 cm^−1^ and 723 cm^−1^ appeared as shoulder. The infrared absorption peaks were similar to the ones identified by Lamontagne *et al.,* [36]. In their study, they identified absorption bands related to asphalt binder of grade of PG 40/50 to include: 2922 cm^−1^ (υ_as_ CH_2_ CH_3_), 2882 cm^−1^ (υ_s_ CH_2_ CH_3_), 1601 cm^−1^ (υ C=C), 1455 cm^−1^ (υ_as_ CH_3_ deformation), 1376 cm^−1^ (υ_s_ CH_3_ deformation), 1031 cm^−1^ (υ SO_2_), 868 cm^−1^, 813 cm^−1^, 747 cm^−1^ (C–H aromatics) and 722 cm^−1^ (alkyl chain).

### 3.3 FTIR analysis of silver nanoparticles

**Figure 2:**
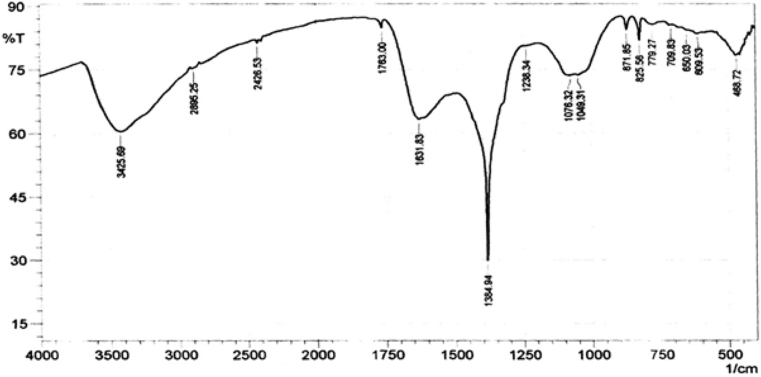
FTIR spectrum of AgNPs (Present study).

The FTIR spectra of AgNPs used in this study were recorded in the spectral range of 4000–400 cm^−1^ as shown in Figure 2. The FTIR spectra of AgNPs were to provide information on possible formation of new bonds or changes in the areas of the peaks related to aging via the modification process. The FTIR spectrum of AgNPs had strong absorption peaks at 3425 cm^−1^, 1631 cm^−1^, 1384 cm^−1^, 1049 cm^−1^ and 1076 cm^−1^. The FTIR absorption peaks were similar to the ones identified by Lateef *et al.,* [23] at 3336 cm^−1^ and 1639 cm^−1^ but differ due to absence of strong absorption peaks at 1384 cm^−1^, 1049 cm^−1^ and 1076 cm^−1^. The band at 3336 cm^−1^ was reported to be associated with the N–H bond of amines, while the 1639 cm^−1^ band reported to be indicative of the C=C stretch of alkenes and the C=O stretch of the carbonyl in amides based on previous studies by Emeka *et al.,* [37] and Shankar *et al.*, [38]. The Ag NPs used in this study and that used by Lateef *et al.,* [23] were synthesized using the pod extract of *Cola nitida*. The AgNPs have been previously shown to have antimicrobial and anti-oxidative properties [23, 24, 25], improved desulphurization of model oil by modified activated carbon [39], and also enhanced anti-oxidative activity and physiochemical composition of *Amaranthus caudatus* [40].

The prominent infrared absorption peaks of AgNPs were as shown in Table 2 and assigned functional groups based on previous results by Lateef *et al.,* [23]; Harish and Renu, [41]; He *et al.,* [42]; Jyoti *et al.,* [43] and Preetha *et al.,* [44].

**Table 2:** Prominent infrared absorption peaks of AgNPs.

Peak, cm^−1^	Bond/Functional Group
3425	N–H bond of amines, O–H stretch and deformation due to water absorption on the metal surfaces
1631	stretching vibration of (NH) C=O group, Ag–O stretching, C=C stretch of alkenes and the C=O stretch of the carbonyl in amides
1384	C–C and C–N stretching in amines
1076	C–N stretch in amines
1049	bending vibration of C–O stretch in amides

### 3.4 FTIR analysis of AgNPs modified base samples

**Figure 3:**
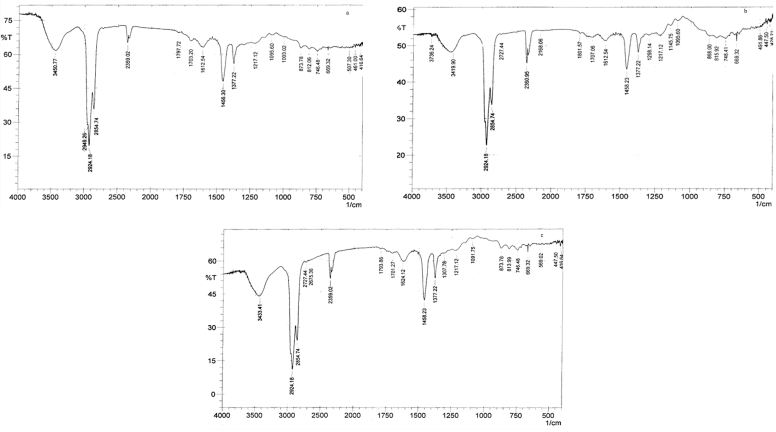
(a) FTIR analysis of 1.5 wt.% AgNPs modified base sample. (b) FTIR analysis of 3 wt.% AgNPs modified base sample. (c) FTIR analysis of 4.5 wt.% AgNPs modified base sample.

The major absorption peaks of infrared spectra of AgNPs modified base samples recorded in the spectral range of 4000–400 cm^−1^ are as represented in Figure 3(a-c). The comparison of peak positions and intensities of various peaks appearing in the infrared spectra of base sample and AgNPs modified base samples showed that there were appearances of additional absorption peaks, indicating the existence of interaction(s) between the AgNPs and the base sample. The additional peaks with wave numbers around 1797 cm^−1^, 1217 cm^−1^and 1300 cm^−1^ were indication of C–C stretch non-conjugated, C–N and C–C stretching in amines, respectively, based on the previous reports by He *et al.* [42], and Jyoti *et al.,* [43]. The sulphoxide peak at 1031 cm^−1^ in the spectrum of base sample disappeared with the appearance of new absorption peak at 1097 cm^−1^ which corresponds to C=O stretch in amides. A progressive reduction in the peak area of the carbonyl peak at 1693 cm^−1^ was evident in a reduction from 3.779 cm^2^ to 2.662 cm^2^, 2.171 cm^2^ and 1.534 cm^2^ due to incorporations of 1.5 wt.%, 3 wt.% and 4.5 wt.% AgNPs, respectively. The possibility of AgNPs dilution/attenuation of the peak was ruled out because low quantity of the AgNPs was added. These are indicators of the anti-oxidative ability of the silver nanoparticles on the bitumen aging process and similarly previous reports by Jyoti *et al.,* [43] have documented the anti-oxidative ability of AgNPs in other media. The AgNPs must have therefore reduced the oxidative susceptibility of the carbon element in the base sample to C=O and as well obliterated the S=O. This anti-oxidative effect was found to be dose dependent. The mechanism of the anti-oxidative action of AgNPs may be by the scavenging of the free radical produced during the oxidative aging process. Petersen and Glaser [45], reported the chemistry of the dual mechanism of asphalt oxidation to involve the fast and slow long term reactions both of which involved the production of reactive free radical. The products of these asphalt oxidation were S=O and C=O compounds [45]. Thus, the anti-oxidative action of AgNPs in the bitumen aging process may be associated with the scavenging of the free radical produced, an action that limits the production of C=O and obliteration of S=O compounds in the thermal aging process. Besides, contrary to the anti-oxidative activities of AgNPs reported in this study, Demirbas *et al.,* [46] in their study biosynthesized red cabbage extract Ag^+^ with AgNPs to show how silver ions (Ag^+^) and AgNPs influence anti-oxidative activity of red cabbage anthocyanin by 2,2-diphenyl-1-picrylhy- drazyl (DPPH) radical scavenging capacity assay and reported that Ag^+^ and AgNPs decreased the anti-oxidative activity of red cabbage anthocyanin towards DPPH.

The new absorption peaks around (1797 cm^−1^, 1217 cm^−1^, 1300 cm^−1^ and 1097 cm^−1^) were indications of structural changes in the base sample and were similar to that obtained from the spectrum of AgNPs used in this study. The intensities of these additional new peaks were found to increase with increasing amount of AgNPs in the modified samples which further supports the hypothesis of dose dependent activities of the AgNPs.

### 3.5 Carbonyl index of thermally aged base and AgNPs modified base samples

**Figure 4:**
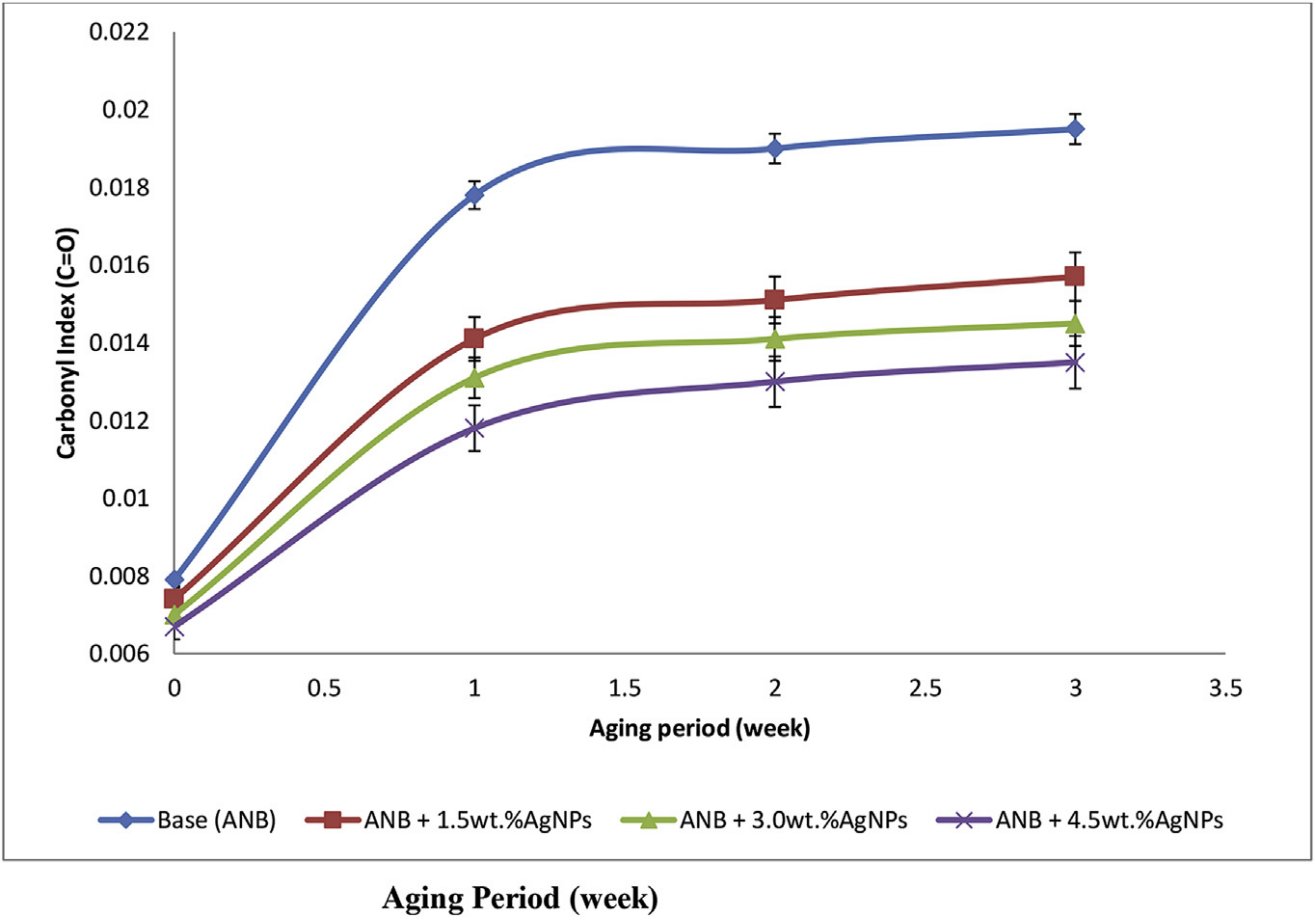
Variation of carbonyl index of Base and AgNPs modified Base samples with period of thermal aging at 60 °C.

The plot of carbonyl indexes of the base and modified base samples against the period of thermal aging at 60 °C is as presented in Figure 4. The values for carbonyl index were found to increase with the aging period for both modified and base samples due to oxidation of the carbon into carbonyl compound i.e. ketone. However, it was found that the values for carbonyl index for the base sample modified with 4.5 wt.% AgNPs were the lowest at various aging period, justifying the anti-oxidative potential of silver nanoparticles and its improvement of base. Thus, the higher the quantity of AgNPs incorporated into the base sample, the higher its anti-oxidative ability.

Carbonyl index only was selected to evaluate the pattern of the AgNPs induced anti-aging behaviour of the bitumen in this study since the anti-oxidative activities of AgNPs have resulted in the disappearance of sulphoxide peak in the modified samples.

### 3.6 Determination of rheological property: complex shear modulus

The complex shear modulus (*G*∗) is a parameter that can be used to evaluate the degree of hardening of the bitumen being total resistance of the binder to permanent deformation when repeatedly sheared [47]. The values of *G*∗ for thermally aged base and AgNPs modified base samples in this study were determined using Oscillatory Disc Rheometer (ODR). They (the values of *G*∗) were determined at lower road pavement temperature (5–25 °C) and higher road pavement temperature (25–70 °C) to determine the resistance of the samples (base and modified base) to fatigue cracking and rutting, respectively.

**Figure 5:**
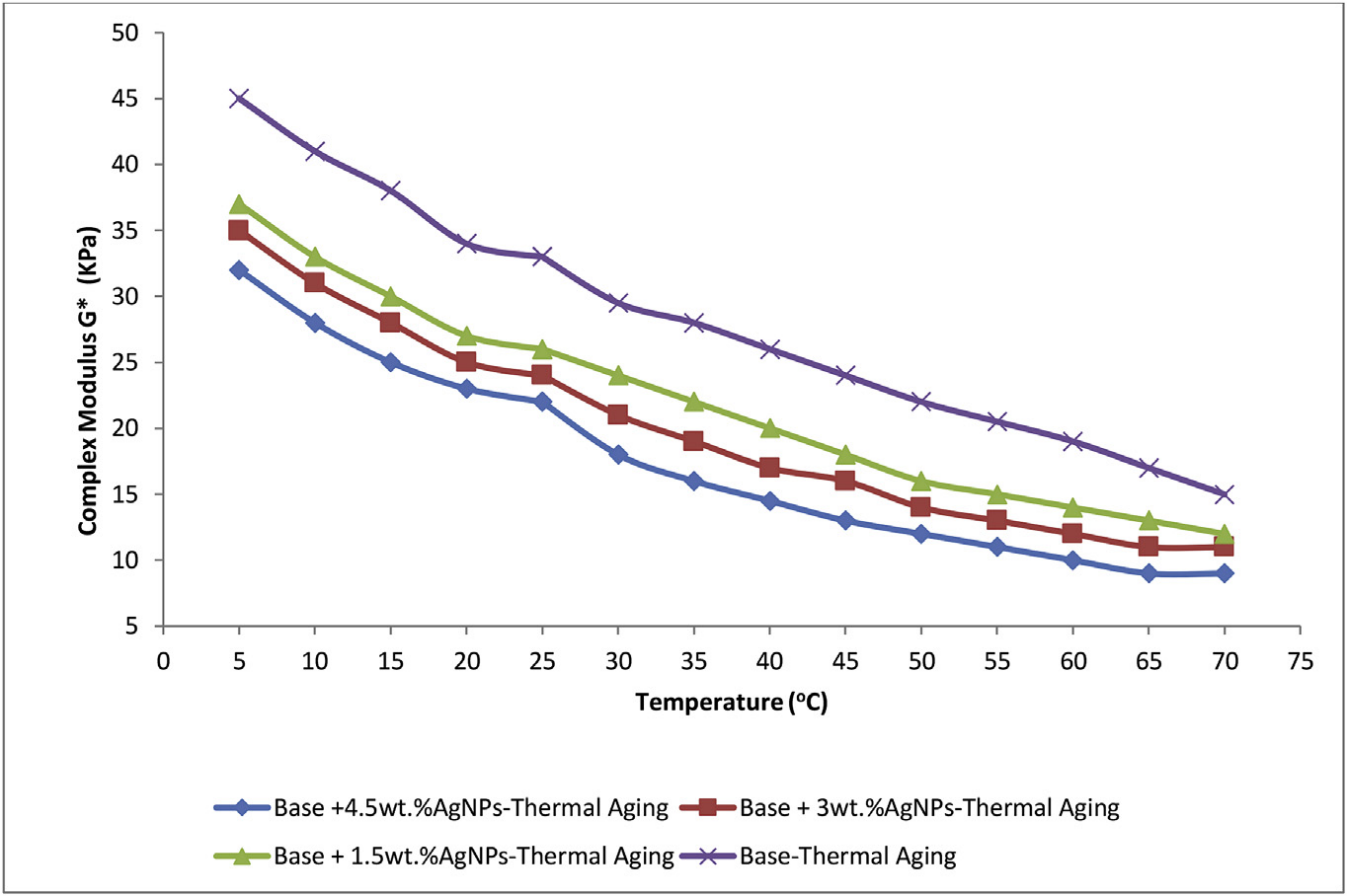
Effect of AgNps composition on *G*∗ as function of temperature (5–70 °C).

The plot of *G*∗ for the aged base and 1.5, 3 and 4.5 wt.% modified base samples at the lower and higher road pavement temperatures are as shown in Figure 5. The base sample was found to be more vulnerable to aging at lower and higher road pavement temperature compared to the modified samples. The vulnerability of base sample to aging at both temperatures has therefore been considered responsible for its higher values of *G*∗ compared to that of modified samples. However, the modified sample with highest quantity (4.5 wt.%.) of AgNPs incorporation has the lowest value of *G*∗ within the temperature (lower and higher) ranges. This implies that the higher the value of G∗ for the aged base sample the lower as the quantity of AgNPs incorporated increased.

The use of ODR in bitumen rheology is a newly conceptualized idea as reported in our previous study [17]. However, effort is being made to come up with how it could be effectively used to determine the phase angle (*δ)* in order to come up with values for rutting parameters. Phase angle is a parameter to indicate an increase or decrease in elasticity of a modified binder compared with the original binder [48]. It is defined as the ratio of *G*∗ to that of sin *δ* [49]. Thus, since the rutting parameters (*G*∗/sin*δ*) is directly proportional to *G*∗ values at higher temperature, it can be deduced after aging from this study that the lower values of *G*∗ for the modified samples at lower and higher road pavement temperatures compared to base sample are indications that the modified samples are less prone to fatigue cracking and rutting, respectively, i.e. the modified samples exhibit a better aging resistance. However, the base sample with highest amount (4.5 wt.%) of AgNPs incorporation showed relatively a better resistance. The severity of the aging effect at higher road pavement temperatures on base is considered to be due to the fact that, the effect of thermal aging results in the breaking of the bond between particles, thus leading to separation between constituent molecules of the base. This lowers the adhesion of the base to the aggregate in road pavement leading to damage due to rutting (long track left on the road as a result of pressure from the car tyres).

Fatigue cracking occurs at lower road pavement temperature due to thermal aging as a result of long term low temperature exposure of pavement which results in physical hardening or rigidity with subsequent fracture of the pavement. Therefore it can be simply inferred that fatigue cracking occurs at lower temperature while rutting occurs at higher road pavement temperature. This agreed with the submission in our previous study by Olabemiwo *et al.,* [17]. The effects of thermal aging on polymer modified ANB samples at lower temperature (for fatigue cracking resistance) and higher temperature (for rutting resistance) were studied. It was deduced that the modified samples showed improved resistance to fatigue cracking and rutting.

Hassan *et al.,* [50] in their study on high and low temperature properties of modified FT-paraffin (Sasobit) bitumen reported that for rutting resistance, a high complex modulus (*G*∗) value is favorable because it represents a higher total resistance to deformation.

### 3.7 Physical and flow properties of base and modified base samples

The results of flow and physical properties of base sample are: Penetration value (82 dmm); softening point temperature (47 °C); Specific gravity (1.047); Flash point (265 °C); Fire point (275 °C); Penetration index (-0.776) and Kinematic viscosity (350 cSt) as reported in our previous study [28].

### 3.8 Effect of AgNPs on base penetration value

**Figure 6:**
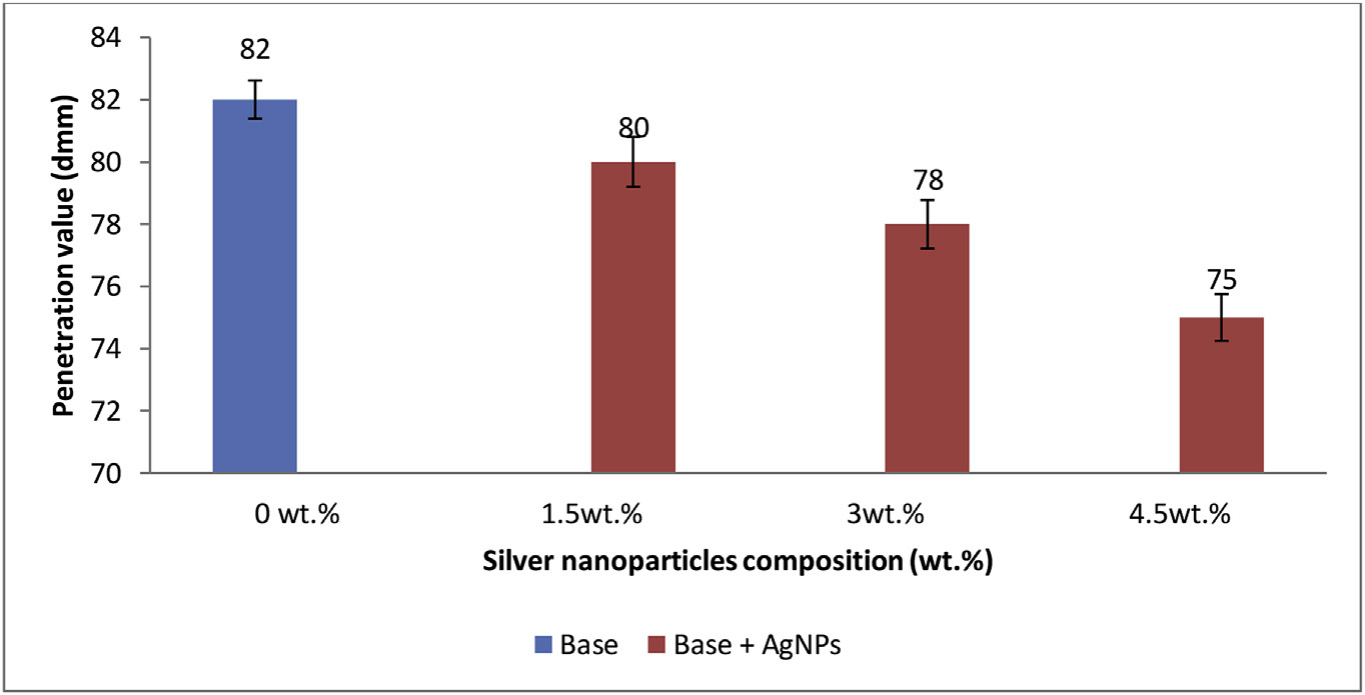
Effect of Ag NPs on Base penetration value.

Penetration is a measure of the consistency or hardness of bitumen [51]. From Figure 6, it can be clearly seen that incorporation of AgNPs caused a decrease in the penetration value of the base sample thereby making it difficult for loaded penetration needle to penetrate the sample. The lower the bitumen penetration value, the higher the load capacity of produced asphalt mixture and the more bitumen softening point. This in turn led to improvement in the stiffness and flexibility of the base sample. Besides, it was observed that as the percentage content of AgNPs incorporated into base sample increased, the magnitude of the penetration value of base sample reduced. It should be noted that the increase in hardness resulting from the incorporation of AgNPs is not that high to make the base fragile thus an improvement. This report corroborated the previous study by Mostafa and Gholamali [51], who modified bitumen with different percentages (0.3%, 0.6%, 0.9% and 1.2%) of Nano SiO_2_ and Nano TiO_2_ and reported a decrease in penetration value of bitumen (68 dmm) to 66 dmm, 63 dmm, 61 dmm, 59 dmm, respectively, for Nano SiO_2_ incorporation and to 65 dmm, 62 dmm, 61 dmm, 62 dmm, respectively, for Nano TiO_2_ incorporation.

### 3.9 Effect of AgNPs on Base Softening point temperature

**Figure 7:**
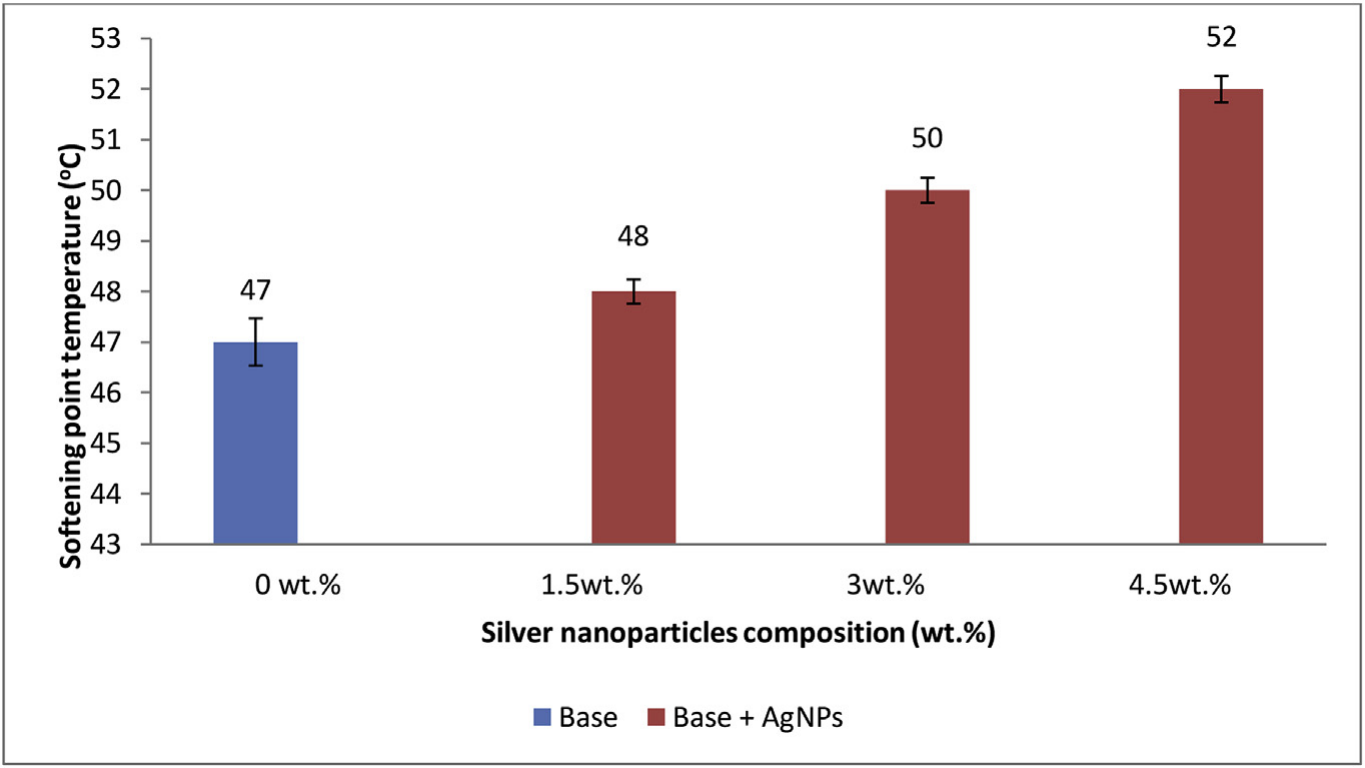
Effect of Ag N-Ps on Base Softening point temperature.

Softening point temperature is the temperature at which the bitumen sample begins to soften [17]. The effects of AgNPs on softening point temperature of base sample are as shown in Figure 7. Addition of AgNPs into base sample caused an increase in the softening point temperature thereby making the base sample less temperature susceptible. However, the increment is dependent of the composition of AgNPs added, implying that the bond between the molecules in the base sample improved as the quantity of AgNPs incorporated increased. Thus, more heat will be needed to break the existing bonds in the base sample. An increase in softening point of base sample on addition of AgNPs is also considered as a reflection that it may offer better rutting resistance at higher road pavement temperature when tested later. The result corroborates the earlier report by Ray and Okamoto [52], and Yao *et al.,* [53]. They reported that by dispersing nano-silica into asphalt matrix one can create polymeric nano composites with enhanced mechanical behaviour, thermal and gas barrier properties. Similarly, Hassan and Siamak [54], modified bitumen with different percentages (1%, 3% and 5%) of Nanosilica and reported an increase in the softening point temperature of bitumen (48.9 °C) to 49 °C, 50.4 °C and 54.8 °C, respectively.

### 3.10 Effect of AgNPs on base penetration index

**Figure 8:**
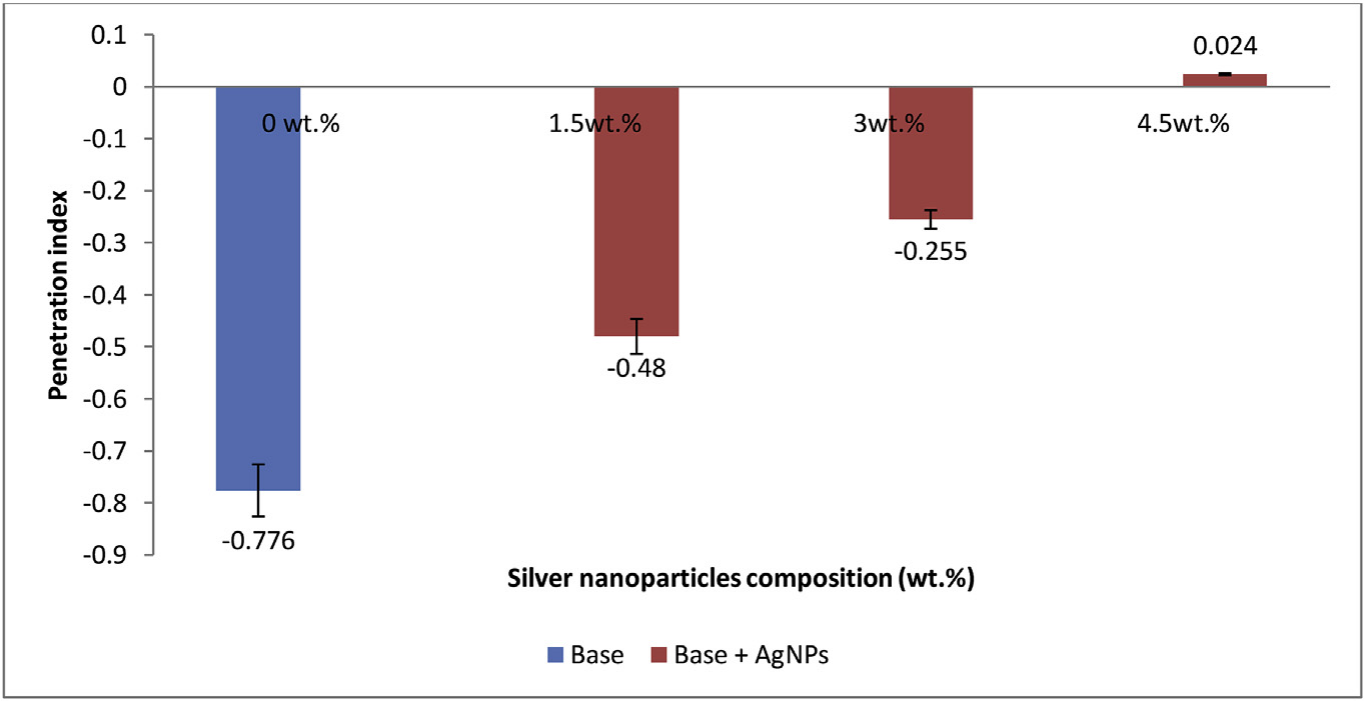
Effect of on AgNPs penetration index of base sample.

Penetration index (PI) is referred to as temperature susceptibility of the modified or unmodified bitumen samples [14]. The AgNPs had an increasing effect on the penetration index of base sample, Figure 8. The penetration index was found to increase as the amount of AgNPs incorporated into base sample increased. This is due to corresponding increase in the softening point temperature and decrease in the penetration value. Noor *et al.* [55], reported in their previous study that bitumen with PI value between +1 and -1 are always considered suitable for road construction. Thus from the results shown in Figure 8, since all the PI values of this study are within the range of +1 and -1, it can also be deduced that all the samples (base and modified base) are suitable for road construction. This report corroborated the earlier report by Hassan and Siamak [54], who modified bitumen with different percentages (1%, 3% and 5%) of Nanosilica and reported an increase in the penetration index value of bitumen (-0.69) to -0.17, -0.56 and -0.14, respectively.

### 3.11 Effect of AgNPs on base kinematic viscosity

**Figure 9:**
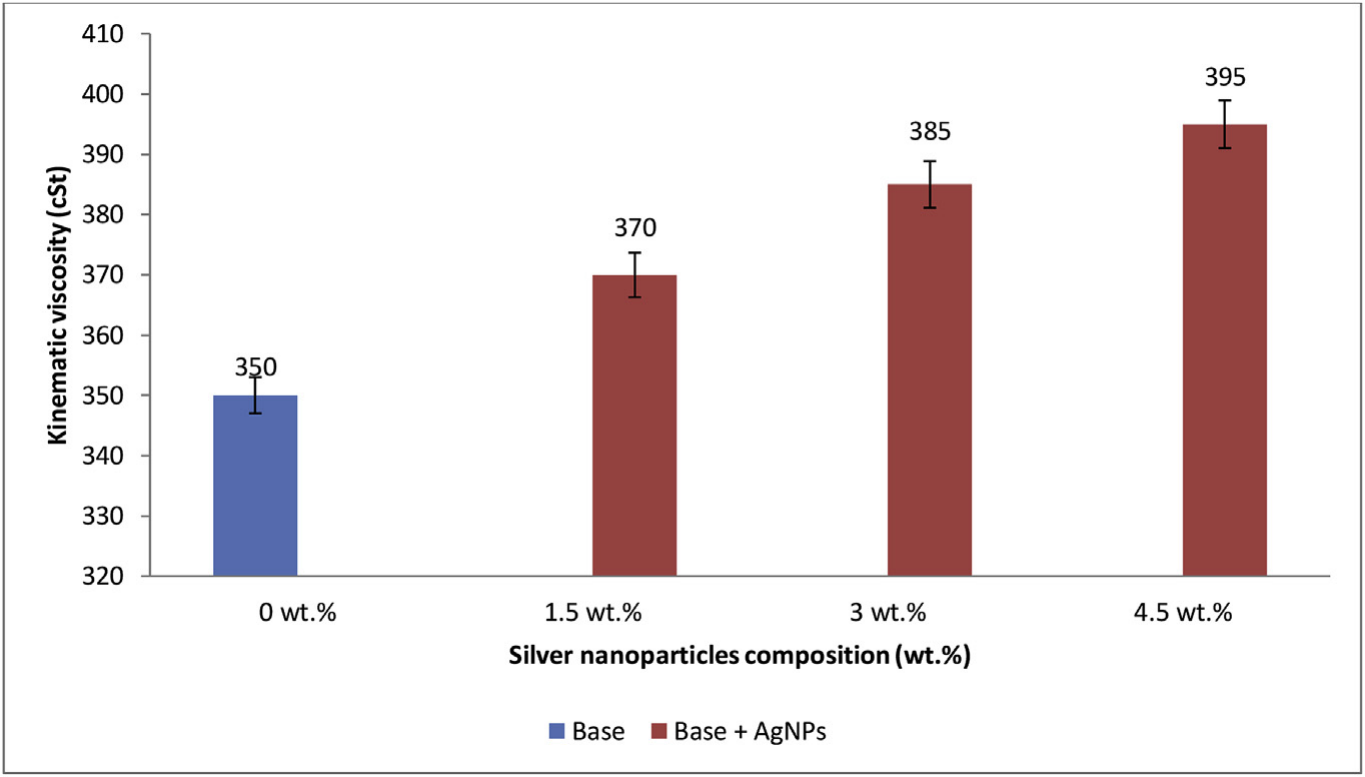
Effect of AgNPs on Base kinematic viscosity.

Kinematic viscosity is a measure of the ease of flow of bitumen at a particular temperature [51]. The results of the effects of AgNPs on kinematic viscosity of base sample are as shown in Figure 9. The addition of AgNPs to base sample raised the value of kinematic viscosity of base sample from 350 cSt to 370, 385 and 395 cSt with 1.5 wt.%, 3 wt.% and 4.5 wt.% of AgNPs incorporated, respectively. The increase is due to the huge surface area, excellent dispersion ability, high absorption, and excellent stability of the AgNPs which led to improvement of bond between the particles in the base sample. An Increase in kinematic viscosity signifies, increase in softening point temperature and decrease in penetration value of the base sample. The increased kinematic viscosity in bitumen at high temperatures can improve the strength of asphalt mixtures against rutting [51]. Zahedi *et al.* [56], modified 64 dmm penetration grade bitumen with different percentages (0.25%, 0.5%, 1% and 1.5%) of carbon nanotubes and reported an increasing value of kinematic viscosity (387 C.st) at 135 °C to 459 C.st, 666 C.st, 1038 C.st and 1482 C.st, respectively.

### 3.12 Effect of AgNPs on base specific gravity

**Figure 10:**
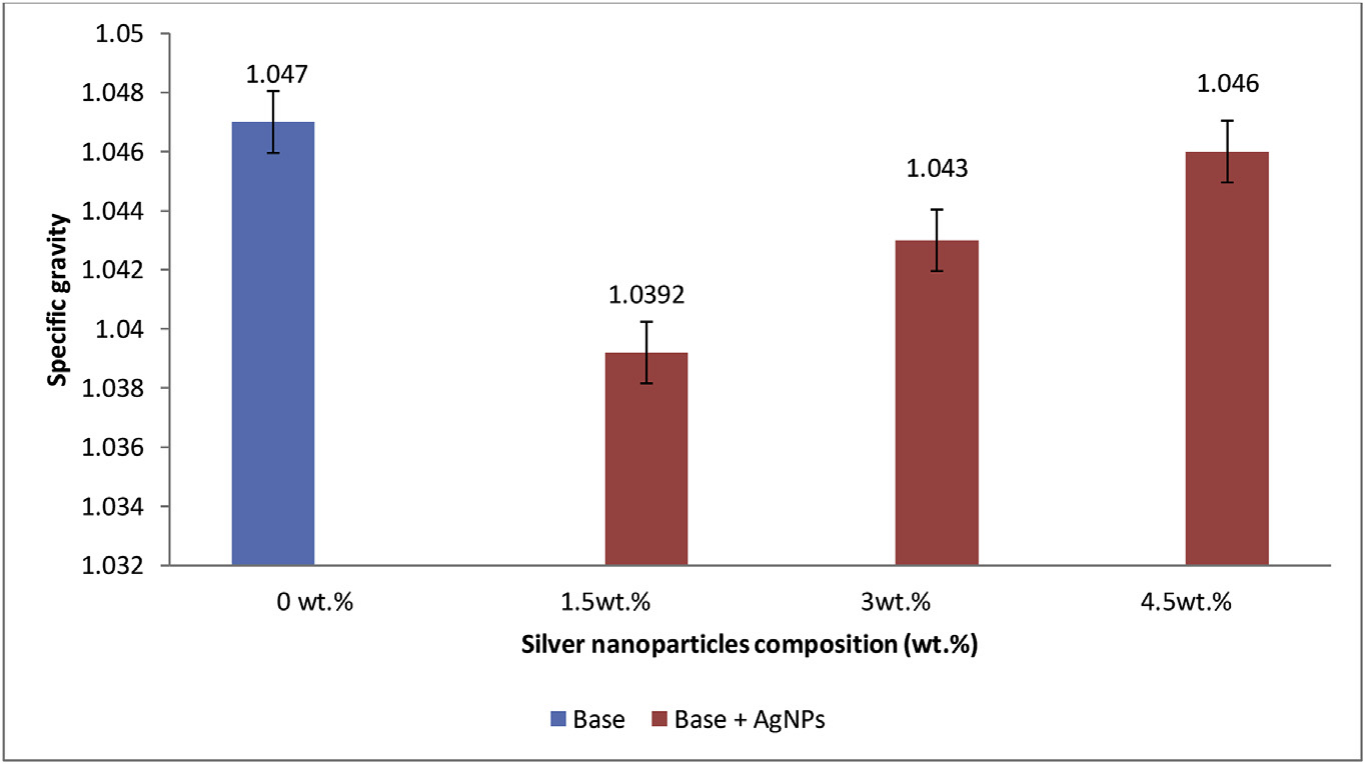
Effect of Ag NPs on base specific gravity.

The plot of specific gravity measured for modified base samples at different AgNPs percentage compositions are as shown in Figure 10. Various percentages composition (1.5, 3.0 and 4.5 wt.%) of AgNPs added to base sample had a slight lowering effect on the specific gravity of base sample. This is due to the specific gravity of AgNPs (0.00017) which is lower than that of the base sample (1.047). This can also be attributed to the fact that when AgNPs was added to base sample, the molecules of AgNPs were swollen up by the maltenic medium of the base sample, thus the volume of the modified sample (total mix) increased. The results obtained showed similarity with the results earlier obtained by Nobinur *et al.,* [57] which investigated the effect of Polyethylene (PE) and Polyvinyl chloride (PVC) on properties of 80/100 penetration grade petroleum bitumen. It was reported that the specific gravity of the binder (1.023) decreased with increasing PE content due to low specific gravity of PE (0.94) but increased with increasing PVC content due to higher specific gravity of PVC (1.25).

### 3.13 Effect of AgNPs on base flash and fire points

**Figure 11:**
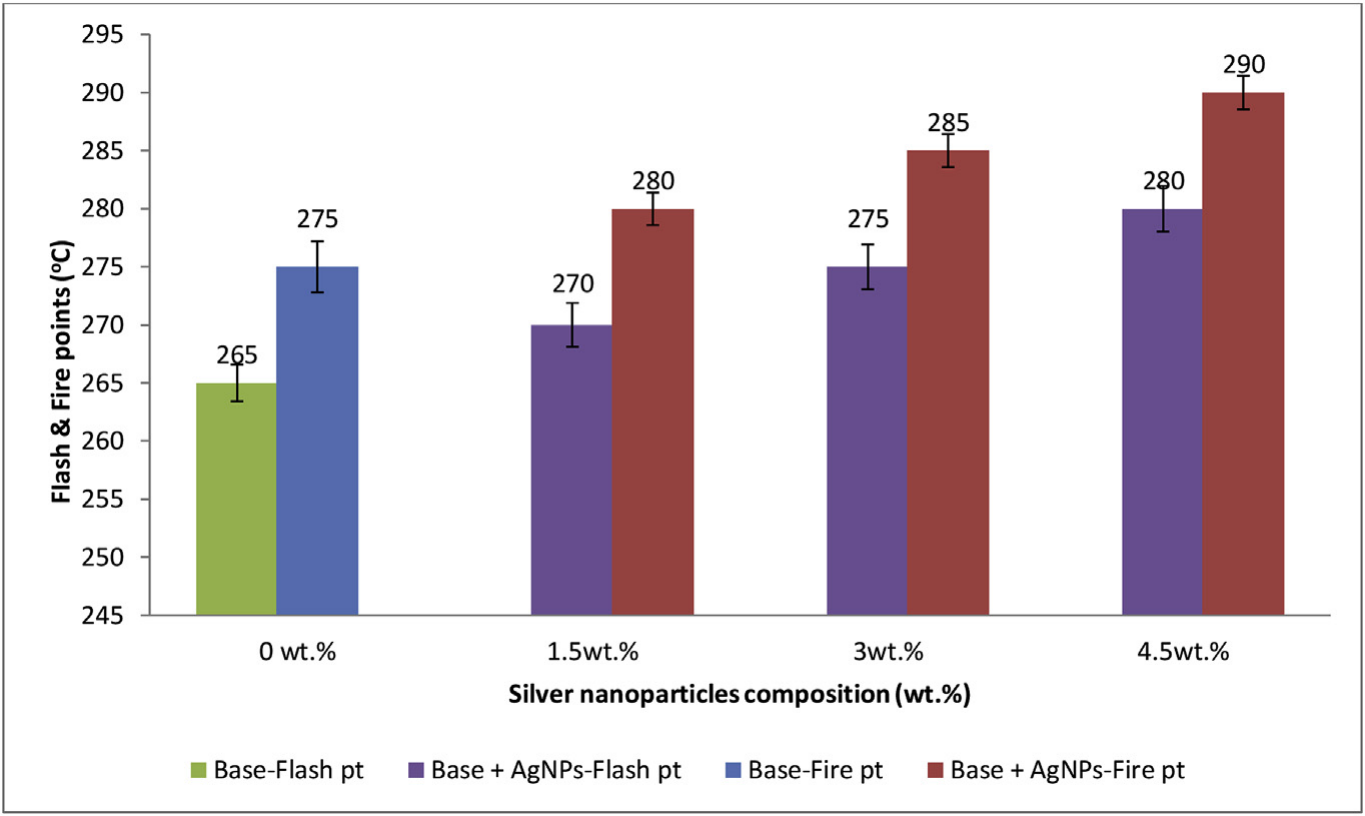
Effect of Ag NPs on Base flash and fire points.

The results of flash and fire points of AgNPs modified base samples at various percentages composition of AgNPs are as shown in Figure 11. The addition of AgNPs into the base sample was found to increase the flash and fire points of the modified samples. Bitumen emitted volatile components which can easily catch fire when heated. However, since the incorporation of AgNPs had been established to increase the softening point temperature of base sample, it implies that more heat will be needed to soften bitumen sample before emission of volatile components. Therefore it can be deduced that, the higher the quantity of AgNPs incorporated into the base sample, the lower is the risk to fire accident during handling i.e. the higher the flash and fire points. Oyedepo and Oluwajana [58], in their study incorporated used tyre in percentage composition which varied from 2-20% into 60/70 Penetration grade bitumen, the incorporation of 20% of the used tyre was reported to increase the flash point of bitumen from 172 °C to 189.2 °C and fire point from 240 °C to 280.152 °C. Similarly the reports of 2, 4, and 6% incorporation of nanosilica into 40 dmm penetration grade bitumen by Pranay *et al.* [59], resulted in increasing flash points from 328 °C to 332 °C, 335 °C and 337 °C, respectively.

## 4 Conclusion

AgNPs was successfully incorporated into base with the aim of investigating its anti-oxidative ability and as well improving the physical and flow properties of the bitumen. The significance of this is to improve the durability of the bitumen in pavement. The anti-oxidative potential of the AgNPs when incorporated into the base sample was noticeable as evident in the disappearance of sulphoxide peak at 1030 cm^−1^ and reduction in the area of carbonyl peak at 1963 cm^−1^ in the spectra of the modified samples. Moreover, there was progressive lowering of the area of carbonyl peak and the corresponding lowering of carbonyl index value which was found to be dependent on the quantity of AgNPs incorporated into the base sample during the modification. This trend was attributed to the anti-oxidative activity of the AgNPs. The mechanism of the anti-oxidative effect of AgNPs was proposed to be due to scavenging of the free radical produced in the oxidation process, an action that limits the production of C=O compounds and led to obliteration of S=O. It was also established from the study that addition of AgNPs led to increase in the softening point temperatures, kinematic viscosities, penetration indexes, flash and fire points and decrease in the penetration values and specific gravities of the base sample. This is because the addition of AgNPs led to increase in the hardness and stiffness of the base sample. It was also found that the effect of AgNPs on base sample is a function of the quantity of AgNPs incorporated.

The ODR test showed that, the modified samples compared to base sample at lower and higher road pavement temperatures are less prone to fatigue cracking and rutting, respectively. However, modified sample with 4.5 wt.% AgNPs incorporation showed comparatively better resistance. This implies that resistance of the modified samples to fatigue and rutting at the lower and higher road pavement temperatures was found to be a function of the quantity of the AgNPs incorporated into the base sample. The AgNPs anti-oxidative activities could therefore be favourable for the improvement of bitumen service life in pavement application.

Although adding AgNPs to bitumen will increase the cost of production, but the reduction in the cost of repair and maintenance of damaged pavement which a modified bitumen will offer is higher enough to compensate for its increase in the cost of production. Thus, the need for modification is justified.

## Declarations

### Author contribution statement

Ojeyemi M. Olabemiwo: Conceived and designed the experiments; Analyzed and interpreted the data; Wrote the paper.

Agbaje Lateef: Conceived and designed the experiments; Contributed reagents, materials, analysis tools or data.

Foluso O. Agunbiade: Analyzed and interpreted the data; Wrote the paper.

S.B. Akanji: Performed the experiments; Contributed reagents, materials, analysis tools or data.

Hassan O. Bakare: Conceived and designed the experiments; Performed the experiments; Analyzed and interpreted the data; Wrote the paper.

### Funding statement

This research did not receive any specific grant from funding agencies in the public, commercial, or not-for-profit sectors.

### Competing interest statement

The authors declare no conflict of interest.

### Additional information

No additional information is available for this paper.
